# Transcriptomic Analysis of *Laribacter hongkongensis* Reveals Adaptive Response Coupled with Temperature

**DOI:** 10.1371/journal.pone.0169998

**Published:** 2017-01-13

**Authors:** Hoi-Kuan Kong, Hon-Wai Law, Xuan Liu, Carmen O. K. Law, Qing Pan, Lin Gao, Lifeng Xiong, Susanna K. P. Lau, Patrick C. Y. Woo, Terrence chi kong Lau

**Affiliations:** 1 Department of Biomedical Sciences, City University of Hong Kong, Kowloon, Hong Kong Special Administrative Region, China; 2 Department of Microbiology and Carol Yu Centre for Infection, The University of Hong Kong, Hong Kong Special Administrative Region, China; National Renewable Energy Laboratory, UNITED STATES

## Abstract

Bacterial adaptation to different hosts requires transcriptomic alteration in response to the environmental conditions. *Laribacter hongkongensis* is a gram-negative, facultative anaerobic, urease-positive bacillus caused infections in liver cirrhosis patients and community-acquired gastroenteritis. It was also found in intestine from commonly consumed freshwater fishes and drinking water reservoirs. Since *L*. *hongkongensis* could survive as either fish or human pathogens, their survival mechanisms in two different habitats should be temperature-regulated and highly complex. Therefore, we performed transcriptomic analysis of *L*. *hongkongensis* at body temperatures of fish and human in order to elucidate the versatile adaptation mechanisms coupled with the temperatures. We identified numerous novel temperature-induced pathways involved in host pathogenesis, in addition to the shift of metabolic equilibriums and overexpression of stress-related proteins. Moreover, these pathways form a network that can be activated at a particular temperature, and change the physiology of the bacteria to adapt to the environments. In summary, the dynamic of transcriptomes in *L*. *hongkongensis* provides versatile strategies for the bacterial survival at different habitats and this alteration prepares the bacterium for the challenge of host immunity.

## Introduction

Rapid adaptation of bacteria to diverse and changing environments required the activation of essential genes for survival and repression of unnecessary or deleterious ones [1]. Gene expression and post-transcriptional regulation are two of the most common modulating strategies in bacteria particularly in response to environmental stimuli such as temperature change or alternation of the habitats, as they provide a quick response for the survival and adaptation to the changing conditions [2]. In the last decade, systematic analysis of transcriptome using RNA-Seq allowed genome-wide quantification of transcripts at various conditions from different habitats, and therefore enhanced our understanding of the bacterial adaptation and survival strategies [3–6].

*Laribacter hongkongensis* is a gram-negative, facultative anaerobic and S-shaped asaccharolytic, belonging to the *Chromobacteriaceae* family of β-proteobacteria [7]. It was firstly isolated and identified from the blood and thoracic empyema pus cultures in an alcoholic liver cirrhosis patient in Hong Kong [8]. Moreover, it was also found in association with community-acquired gastroenteritis and traveler’s diarrhea, and is likely to be globally distributed as suggested by the travel histories from patients [9–12]. Apart from acting as infectious agent in human, *L*. *hongkongensis* is also capable to infect zebrafish through dermal abrasion or intraperitoneal injection. Infected zebrafish died in dose-dependent manner with distinguishing clinicopathological characteristics such as lethargy and abdominal hemorrhage. These features supported the Koch’s postulates for *L*. *hongkongensis* to be an fish infectious agent [13]. *L*. *hongkongensis* has been widely found in intestine (60%) from commonly consumed freshwater fish of the carp family, drinking water reservoirs and Chinese tiger frogs in Hong Kong as well as little egrets in Hangzhou [14–16]. Nevertheless, the capability to adapt to different environmental conditions that guarantees the survival of *L*. *hongkongensis* in diverse host and habitats is still largely unknown.

Previously, we sequenced the genome of *L*. *hongkongensis* (HLHK9) and identified unique genetic elements that are responsible for surviving under various external stimulations such as pH, osmotic pressure and oxidative stress [17–19]. Moreover, proteomic and gene regulatory studies of two *arc* gene cassettes as well as two copies of the *argA* and *argB* genes in *L*. *hongkongensis* at two physiologically relevant temperatures (37°C for human body temperature and 20°C for freshwater habitat temperature) revealed its acid resistance mechanisms under anaerobic and acidic conditions [19–22]. Although a number of evidence displayed the complex and versatile adaptation to different body temperatures of niches, systematic transcriptomic studies have not yet been carried out. Here we present the global transcriptomic analysis of *L*. *hongkongensis* using RNA-Seq at the temperature of 20°C and 37°C, mimicking the body temperatures of fish and human. We identified several important pathways involved in the adaptation of *L*. *hongkongensis* to the shift of metabolic equilibriums and over-expression of stress-related proteins. Moreover, these temperature-induced gene expressions adjusted the physiology of bacteria in order to survive at different habitats. In conclusion, our findings provided insight into the bacterial adaptation to the host temperature, which may ultimately contribute to the prevention and treatment of *L*. *hongkongensis* infection.

## Materials and Methods

### Bacterial strain and growth conditions

*L*. *hongkongensis* HLHK9, a clinical isolate from a Hong Kong patient who had severe gastroenteritis, was shared from Patrick Woo’s Lab. Bacteria strain was grown in brain heart infusion (BHI) or BHI agar at either 20°C or 37°C with shaking at 250 rpm. Bacterial cell density was measured by optical density at 600 nm.

### Growth curve measurement

*L*. *hongkongensis* was cultured in 100mL fresh BHI broth at 20°C or 37°C. The optical density at 600 nm (OD_600_) of the culture was measured by Spectrophotometer (UV mini-1240) every 1 hour until it reached stationary phase. The growth curve was plotted using time as x-axis and absorbance at 600 nm as y-axis. The absorbance at each time point was calculated by taking the mean of triplicate value. Doubling time of bacteria was determined by period of time to grow from exponential phase to stationary (OD_600_ 1 to OD_600_ 2).

### RNA extraction, library construction, RNA sequencing and data analysis

Cells at mid-log phase (OD_600_~1.2) were harvested and subjected to total RNA extraction for RNA sequencing. Briefly, 50 mL cell pellet was lysed with 2 mL TE buffer containing 20 mg/mL lysozyme (Sigma) and 0.4 mg/mL Proteinase K solution (Ambion) for 10 minutes. The cell suspension was further lysed with TRIzol reagent (Invitrogen) for 5 minutes. Cell lysate was then mixed with chloroform; total RNA was separated from aqueous phase and precipitated with isopropanol (Sigma). Extracted RNA was treated with TURBO DNase (Ambion) and purified using acidic phenol-chloroform (Ambion) to remove genomic DNA. The RNA was then subjected to MICROBExpress Kit (Ambion) and Ribo-Zero rRNA Removal Kits (Epicentre) to eliminate rRNA according to manufacturer’s instructions. The RNA quality and quantity were monitored by Bioanalyzer (Agilent) using RNA 6000 Pico Kit (Life Technologies). The library was constructed with Ion Total RNA-Seq Kit v2 (Ambion) according to manufacturer’s instructions. The purified RNA was next sequenced using Ion Torrent sequencing platform on Ion 318 Chips (Life Technologies).

Raw sequence data with 5% unknown nucleotides or in low abundance at two temperatures were filtered to reduce false positives brought by random errors. We adopted Phred-scaled base quality scores (Q20) to minimize incorrect bases since this cutoff scores provided Base Call Accuracy up to 99%. Processed reads were mapped onto *L*. *hongkongensis* HLHK9 genome (GenBank accession: NC_012559.1) using TMAP [23–27] with default parameters. The mapped sequencing reads were then visualized in Integrated Genome Viewer (IGV) [28, 29]. Gene expression was normalized to RPKM (Reads Per Kilobase of transcript per Million mapped reads) from reads that mapped on the gene strand within 175 bp prior to start codon to 75 bp after stop codon. RPKM = 10^9^R/NL that R is the number of reads mapped, N is the total number of mapped reads, and L is the length of gene region. The sequencing data has been deposited in NCBI Sequence Read Archive (SRA) database (accession number: SRR5053677 and SRR5053678).

### Quantitative Real-Time PCR (qRT-PCR)

Total RNA was extracted using RNeasy Mini Kit (Qiagen), followed by TURBO DNase (Ambion) treatment and incubated with inactivation reagent (Ambion) to inactivate DNase. One μg treated RNA was reverse transcribed into cDNA by Superscript III First-Strand Synthesis System (Life Technologies). Primers for qRT-PCR were designed with Primer3 software [30] and listed in S1 Table with *rpoB* as the endogenous control. The PCR was run on a 7500 Fast Real-time PCR System (ABI) with a default program as 50°C 2 min, 95°C 10 min for 1 cycle, and 95°C 15 s, 60°C 1 min for 40 cycles. The relative expression level (REL) were calculated by Ct values from all qRT-PCR reactions in triplicate using formula 2^-△△Ct^.

## Results and Discussion

### Genome-wide analysis of transcriptome at 37°C and 20°C

**Fig 1:**
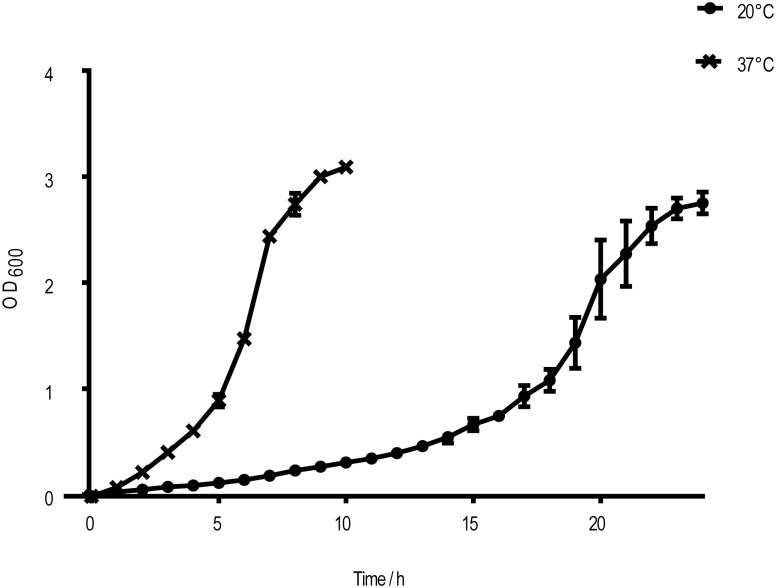
The growth curve of L. hongkongensis at 20°C and 37°C. Vertical error bars represented standard deviation of biological triplicate.

We performed transcriptome sequencing to compare gene expression of *L*. *hongkongensis* grown at two physiological temperatures. In our RNA-seq, total reads were 5,391,268 and 5,142,669 at 37°C and 20°C respectively. The average read lengths were 108 bp and 112 bp at 37°C and 20°C respectively. In order to evaluate the influence of temperature in growth kinetics, we measured the optical density at 20°C and 37°C (Fig 1). The doubling time was 1 hour and 15 minutes at 37°C and extended to 3 hours and 15 minutes at 20°C, indicating the growth rate was two times faster at 37°C. Bacteria on the same logarithmic phase (OD_600_~1.2) at both temperature were collected for RNA-seq. Differentially expressed genes that were in low abundance at both temperatures were removed. More than 90% read counts were qualified and mapped to the genome of *L*. *hongkongensis* (HLHK9, accession number NC_012559.1). According to the mapping result (S1 Fig), more than 70% of reads were mapped to the coding region. Intriguingly, 10% of total reads were mapped to regions that are either intergenic regions or anti-sense to the coding regions, and they are possibly the non-coding or small RNAs.

**Fig 2:**
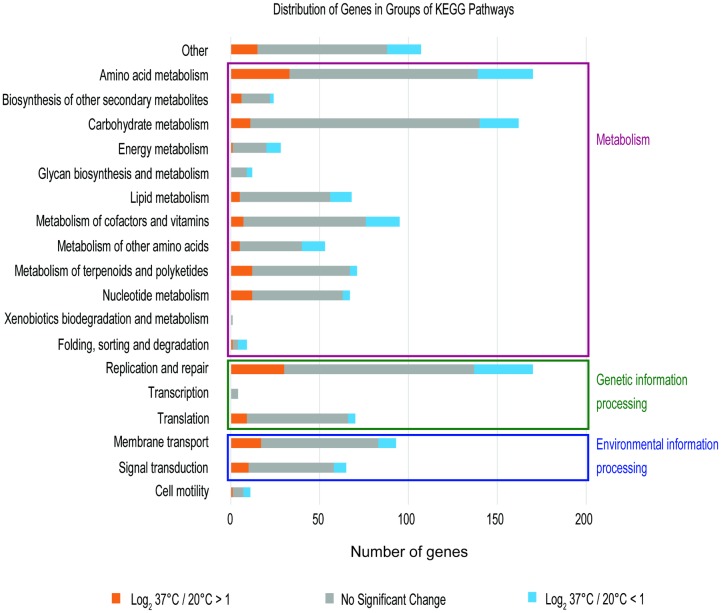
Gene categories distribution of differentially expressed genes in KEGG pathway at 20°C and 37°C. Bars represented the abundance of genes up-regulated at 20°C (blue), 37°C (orange) and no expression difference (grey) in each category.

To define the differential gene expression between two temperatures, we set the threshold of fold change as |log_2_ (37°C/20°C)| > 1. Subsequently, 363 and 482 genes were identified as up-regulated genes at 20°C and 37°C respectively, which accounts for 11% and 15% of total genome in *L*. *hongkongensis*. The abundance of differential expressed genes was similar to other genome-wide studies of bacteria that approximately 10% of genes are responsible for temperature adaptation in bacteria [31]. We further categorized the distribution of differentially expressed genes based on their functions and pathway in Kyoto Encyclopedia of Genes and Genomes (KEGG) [32] as shown in Fig 2. Overall, genes involved in carbohydrate, energy and lipid metabolic pathways, and cofactors, vitamins and other amino acids metabolism were upregulated at 20°C than those at 37°C. On the contrary, higher number of elevated gene expression in metabolism of terpenoids and polyketides, nucleotide metabolism, membrane transport and signal transduction pathways were observed at 37°C compared with 20°C. Indeed, more than 50% of genes in every functional pathway showed no significant changes which indicated that the bacteria often keep the minimum disturbance to transcriptomic equilibrium in response to external stimuli and it will prevent the cell from causing severe energy consumption and any harmful side effect [31].

In order to further confirm the accuracy of RNA-seq data, we performed the quantitative RT-PCR (qRT-PCR) to validate several genes that showed differential expression in three biological samples. Genes in different metabolic pathways with log2 (37°C/20°C) between ±1 and ± 4 in RNA-seq were selected for validation. As shown in S2 Fig, differential gene expression between RNA-seq and qRT-PCR showed positive correlation (R^2^ > 0.9) in the linear regression analysis among 17 genes, suggesting the changes obtained in our RNA-seq were consistent and reproducible in biological samples.

In S2 Table, we also compared the consistency of changes between transcripts level in RNA-seq and protein level from our previous proteomic study [20]. We found that more than 70% of the genes (8 out of 11 genes, 72%) showed the same regulatory pattern in both transcriptomic and proteomic studies. This positive correlation indicated similar expression level changes in both RNA and protein of *L*. *hongkongensis* after the stimuli of the environmental changes.

**Fig 3:**
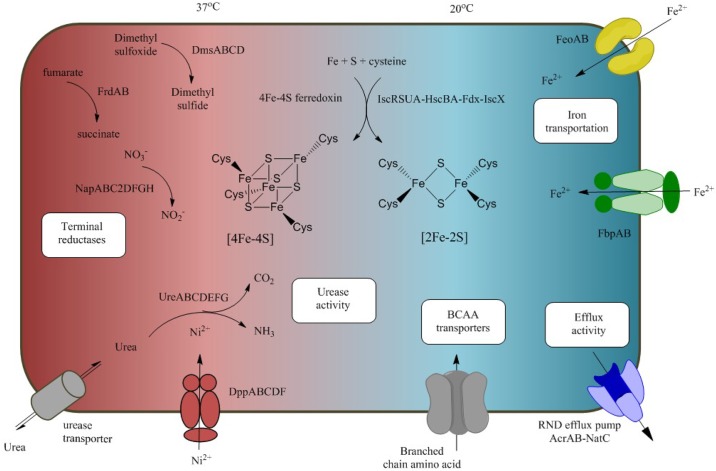
Model of temperature-induced pathways involved in host pathogenesis. Gene operons induced at 20°C and 37°C were drawn in blue and red region respectively.

Overall, we identified numerous genomic cassettes including urease biosynthesis, terminal reductase, iron acquisition and efflux activity, in which their gene expression levels were coupled to particular temperatures. Under the same scale, genes in these cassettes were preferentially expressed at either 20°C or 37°C, suggesting the important role of the particular pathways involved in the adaptation of *L*. *hongkongensis* to different habitats. A model describing the temperature-induced gene network was shown in Fig 3.

### Differential expression pattern of *L*. *hongkongensis* in response to temperature stress

**Fig 4:**
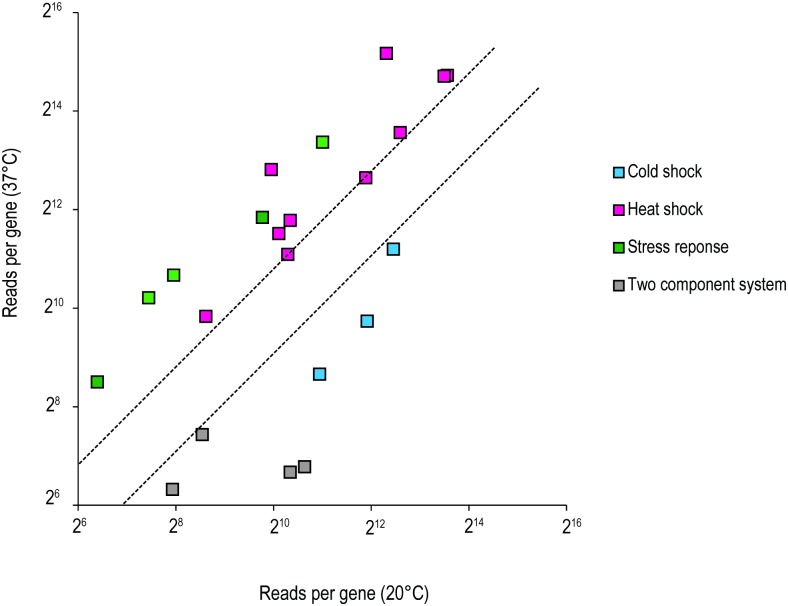
The relative expression of genes known to be related to heat shock, cold shock, stress response protein and two component systems were compared at 20°C and 37°C by RNA-seq. Each square represents for a single gene, is categorized according to its function. The dashed lines represent a 2-fold over-expression at 37°C (upper line) and 20°C (lower line) conditions.

*L*. *hongkongensis* was often found in various hosts which live at different temperatures such as fishes, frogs and human, indicating the versatility of this bacterium to change its physiology in adapting different conditions. To cluster the proteins of particular pathways or functions with differential expression patterns, we plotted the graphs of expression levels at two different temperatures in a log scale (Fig 4). The protein groups above or below the dot lines represented the high expression level at 37°C or 20°C respectively whereas those inside the two dot lines had no significant changes between two temperatures. As shown in Fig 4, the drastic changes in temperature induced the cold and heat shock response systems to adjust the physiological equilibrium of bacteria for adaptation. For example, the cold shock related proteins including HscA, HscB (LHK_02861, LHK_02862) and the cold shock transcription factor CspA (LHK_00932) were upregulated (2.4 to 4.9-fold) at 20°C whereas the expression level of heat shock proteins such as DnaJ/DnaK/GrpE chaperone complex (LHK_02738-LHK_02740), GroEL-GroES chaperonin complex (LHK_00659-LHK_00660) and HslV/HsIU protease (LHK_00004, LHK_00005) were elevated (1.7 to 2.7-fold) at 37°C. Moreover, we found that the homologous of stress protein UspA (LHK_00487, LHK_00590) and several stress response proteins (LHK_01454, LHK_01835, LHK_03102) were upregulated dramatically at 37°C (4.2 to 6.8-fold). UspA is one of the universal stress proteins that extensively studied, and is always induced by a plethora of environmental stressors such as starvation and high temperature in order to protect the organism [33, 34]. Intriguingly universal stress proteins were demonstrated to play role in the actions of adhesion, motility and biofilm formation in *Escherichia coli* [35] as well as the persistence of *Mycobacterium tuberculosis* within human host [36], indicating the induction of USP at human host temperature not only regulates genes in various cellular pathways to protect bacteria under extreme temperatures but also increases the virulence and become more invasive during infection. In *L*. *hongkongensis*, the upregulation of USP at 37°C possibly utilizes the similar strategy to activate the virulence factors and enhance the pathogenicity during human infection.

Apart from cold and heat-shock protein, two-component signal transduction systems (TCSs) is also essential to sense and respond to various external conditions in bacteria. The systems consist of histidine kinase for sensing external stimulus and the corresponding regulator for altering gene expression to adapt to external stress [37]. In our RNA-seq, we found that some TCSs including PhoB/PhoR and LHK_02501/LHK_02502 were upregulated at lower temperature. As shown in Fig 4, 3-fold enhancement of Pho regulon including sensor *phoBR* and the phosphate—specific transporter *pstBACS* operon were observed at freshwater temperature. Histidine kinase PhoR phosphorylates the corresponding regulator PhoB according to external phosphate concentration to activate transcription of Pho regulon including *pstBACS* operon and overexpress PstBACS protein. The PstBACS protein then acts as a phosphate-specific transporter for transferring inorganic phosphate from periplasm to cytosol under phosphate starvation [38]. Since aquatic environment is generally considered as lacked of phosphate [39], stimulation of *pstBACS* operon may assist the bacteria for survival. In addition, Pho regulon was reported to modify lipid A and fatty acid composition [40]. Interestingly, several genes related to the metabolic process of peptidoglycan, which is the major component of cell wall, including *mtlD*, *mrcA* and *slt* were also upregulated at 20°C. Maintaining membrane integrity and fluidity is one of the adaptation strategies during cold shock [41, 42]. The activation of Pho regulon and upregulation of peptidoglycan metabolism possibly modify the membrane lipid composition and ante-iso/iso branching pattern to retain membrane integrity at lower temperature.

Two component systems have been shown to play significant roles for cold tolerance in bacteria [43]. The DesK/DesR proteins of *Bacillus subtilis*, for example, can induce genes for membrane fluidity modification at low temperature [44]. LHK_02501/LHK_02502 are proteins containing histidine kinase and response regulator receiver domain, integrating as a putative two-component system. Upregulation of this regulon at 20°C (12 and 14 fold respectively) in our result suggested the possible function of cold-induced transcriptional regulator in *L*. *hongkongensis*.

### Temperature-regulated dynamic physiological switching in *L*. *hongkongensis*

**Fig 5:**
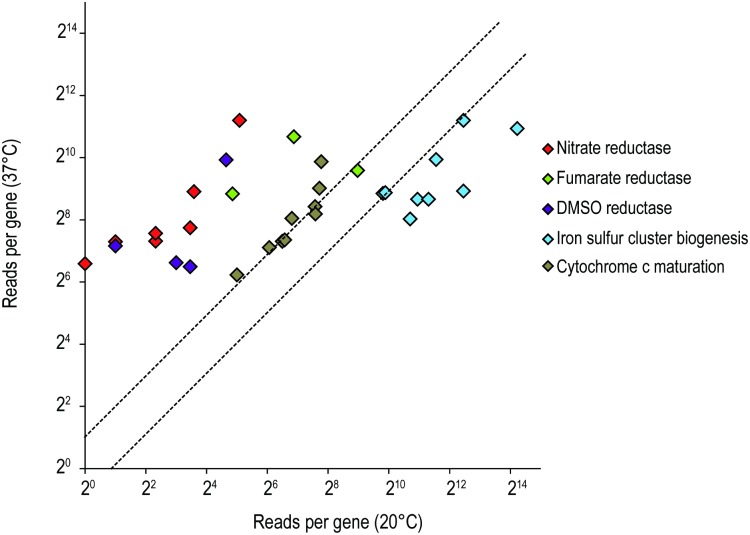
The relative expression of genes known to be related to oxidoreductase genes were compared at 20°C and 37°C by RNA-seq. Each rhombus represents for a single gene, is categorized according to its function. The dashed lines represent a 2-fold over-expression at 37°C (upper line) and 20°C (lower line) conditions.

In bacterial respiration, electrons are transported from electron donor to terminal electron acceptor across cytoplasmic membrane to generate a proton gradient. During aerobic respiration, oxygen is the main electron acceptor to be reduced to water via cytochrome o or cytochrome d oxidases [45]. On the other hand, bacteria reduce other electron acceptors such as nitrate, nitrite, DMSO and fumarate into nitrite, ammonium, DMS and succinate respectively during anaerobic respiration [46]. In our transcriptome analysis, several anaerobic respiration related enzymes were upregulated significantly at 37°C, in particular the expression of anaerobic reductase. As shown in Fig 5, 20-fold enhancement of nitrate reductases (LHK_02079-LHK_02085) and more than 10-fold increase of fumarate reductases (LHK_02340-LHK_02342) and dimethyl sulfoxide reductase operons (LHK_02496- LHK_02499) were observed at 37°C compared with 20°C. The upregulation of these terminal reductases at 37°C probably due to the decreased solubility of oxygen at higher temperature. In fact, *L*. *hongkongensis* is often found in human intestine where the oxygen level is very low. Therefore, induction of anaerobic respiration-related genes at 37°C possibly support the bacteria to adapt to hypoxia condition during infection for survival. Apart from anaerobic reductase, Class III ribonucleotide reductase NrdD and NrdG (LHK_02258- LHK_02259) were dramatically up-regulated (73 and 91-fold) at 37°C. Since these proteins catalyze the reduction of ribonucleoside triphosphates to deoxyribonucleotides in anaerobic condition, they may also play roles in providing sufficient deoxyribonucleotides for bacteria to maintain a faster DNA synthesis and repair systems at 37°C. On the other hand, the genes involved in iron-sulphur (Fe-S) cluster biogenesis were highly expressed at 20°C compared with 37°C (Fig 5). Iron-sulphur clusters are fundamental to numerous biological processes, in particular acting as protein cofactors for respiration, DNA replication and repair, and gene regulation in bacteria [47]. Genes associated with iron homeostasis could enhance *L*. *hongkongensis* to overcome the stress caused by lower temperature.

### Regulation of temperature-associated virulence and pathogenic factors

**Fig 6:**
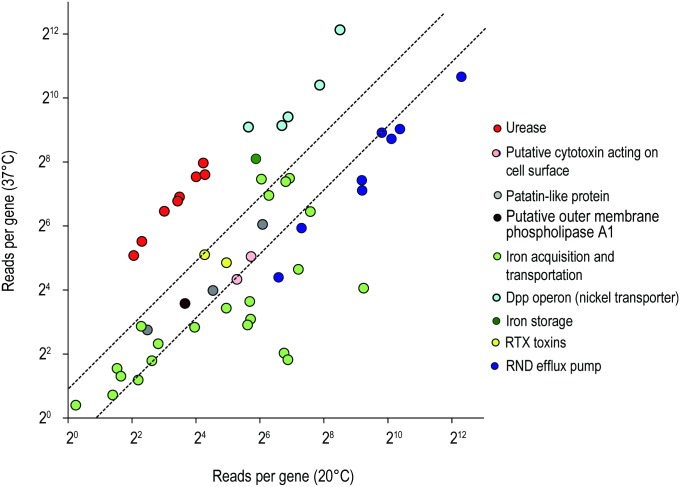
The relative expressions of genes known to be related to pathogenic factors were compared at 20°C and 37°C by RNA-seq. Each dot represents a single gene and is categorized according to its function. The dashed lines represent a 2-fold expression at 37°C (upper line) and 20°C (lower line) conditions.

To understand the pathogenesis of *L*. *hongkongensis*, we also compared the gene expressions of virulence factors between two temperatures (Fig 6) and found that most of the iron acquisition and transportation genes but not iron storage genes were upregulated at freshwater temperature (20°C). For example, the transcription of ferrous iron transporter *feoAB* (LHK_03044 and LHK_03045), TonB-dependent receptor (LHK_00497, LHK_01193) and transferrin transports homolog *fbpAB* (LHK_02634-LHK_02636) increased significantly (2 to 40-fold) at 20°C. Iron is vital mineral for bacteria to maintain energy generation and protect bacteria against oxidative stress [48–50]. Since iron is essential in metabolism, hypoferremia greatly restricts the growth of pathogens and thus countermeasures are taken to evade iron-deplete condition in bacteria [51]. Secreting siderophore is one of the efficient methods to acquire iron from host. Siderophores are small iron chelators with extremely high association constant. Such strong iron binding affinity surpasses host transferrin binding [52]. Some pathogens utilize receptors to scavenge iron directly from transferrin and lactoferrin to overcome iron limiting defense system [53, 54]. In *L*. *hongkongensis*, however, no siderophore was found. Instead, homolog of TonB-dependent siderophore receptor (LHK_00497) and ABC transporters for transferrin and lactoferrin are presented, suggesting that iron is acquired through these iron binding proteins [18]. Together with the over-expression of Fe-S cluster described above, the increase of genes expression involved in iron acquisition suggested that *L*. *hongkongensis* obtained iron through multiple pathways to maintain iron homeostasis which in turn, play roles in the pathogenesis of fish infection at freshwater temperature. Noteworthy, iron sequestering systems were often found to be temperature-regulated and overexpressed at temperatures below that of their optimal growth (TBO) in other fish pathogens, which are similar to other virulence factors such as bacteriolysis-related proteins and secretion systems [55].

### Urease and nickel transport operons were upregulated at human body temperature

Previously, we demonstrated that arginine deiminase pathway was far more important than urease for acid resistance and intracellular survival in *L*. *hongkongensis*, which is different from other gastrointestinal tract pathogens [56]. In our transcriptomic analysis, intriguingly, the mRNA levels of urease and urea transporter (LHK_01035-LHK_01037, LHK_01040- LHK_01044) were enhanced significantly (6 to 12-fold) at 37°C. Moreover, the ABC transporters for nickel including *dppA* and *dppBCDF* (LHK_00667, LHK_00939–00942) which provide nickel as cofactor to catalyze urea hydrolysis into ammonia to neutralize the acidic gastrointestinal environment [17], were also increased more than 5-fold as well (Fig 6). Since the functional consequence of urease pathways is to assimilate urea as nitrogen sources for amino acid production [57], utilization of urea by increasing expression of urease cassette and the nickel transporting genes could be the potential alternative energy sources for *L*. *hongkongensis* to grow at 37°C. Indeed, ureases in *Klebsiella* and *Proteus* were shown to induce ammonia and lipopolysaccharide production which could eventually lead to hepatic encephalopathy [58, 59], syndrome that often found in liver cirrhosis patients. Since *L*. *hongkongensis* was firstly identified in alcoholic liver cirrhosis patients, understanding of the association of ureases pathways to liver dysfunction could lead us to the insight of the pathogenesis of *L*. *hongkongensis*.

### Branched-chain amino acid transporter was enhanced at freshwater temperature

**Fig 7:**
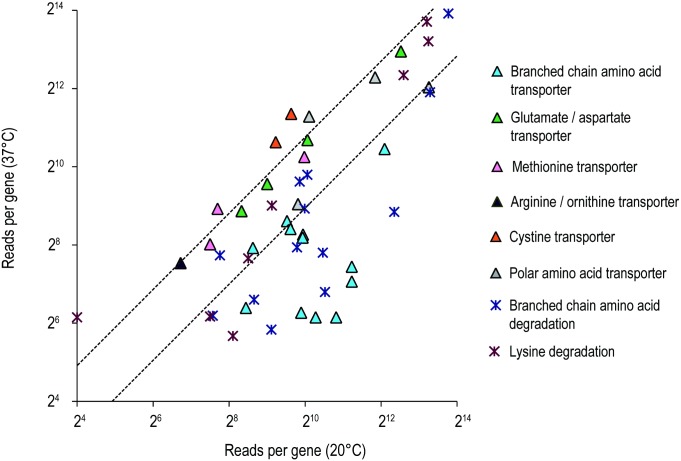
The relative expressions of genes known to be related to amino acid transporters and degradation were compared at 20°C and 37°C by RNA-seq. Each triangle or cross represents for a single gene. The dashed lines represent a 2-fold over-expression at 37°C (upper line) and 20°C (lower line) conditions.

Branched-chain amino acid (BCAA) leucine, isoleucine and valine are essential low-melting-point fatty acid for bacterial survival, in particular gram-positive bacteria [60]. Interestingly, we noticed that the gene expression of BCAA biosynthesis were similar at both temperatures but those related to BCAA degradation and transportation system were elevated significantly at 20°C (Fig 7). Bacteria normally acquire BCAAs either through BCAA transporters or by intracellular synthesis. In addition, certain bacterial BCAA transporters including *Pseudomonas putida* [61] and *Shewanella piezotolerans* [62] were found to be temperature regulated. Degradation and biosynthesis of BCAA were proposed to generate precursors for Branched-chain fatty acids (BCFA) in the BCFA synthesis model of *Shewanella* under different external temperatures. The upregulation of BCAA transporters at cooler temperatures was proved to transfer exogenous BCAA into cell for BCFA synthesis. Therefore, promoting BCFA portion could help to maintain the membrane fluidity of bacteria, which is a conventional strategy in cold adaptation [63]. The induction of BCAA transportation system in *L*. *hongkongensis* possibly share similar function of that in *Shewanella*.

### RND efflux activity was upregulated at 20°C

**Fig 8:**
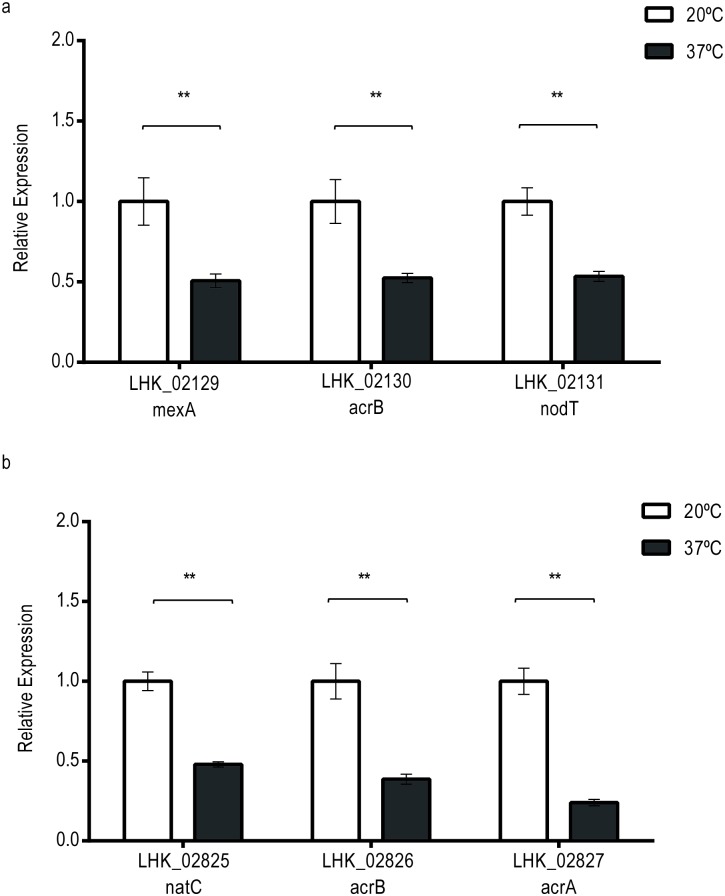
The graphs showed the mRNA expression of a) LHK_02129–02131 b) LHK_02825–02827 at 20°C and 37°C. Vertical error bars showed the standard deviations of biological triplicates. Expression level at 20°C was taken as reference for comparison. *P-value<0.05; **P-value<0.01.

Previous evidence showed that *L*. *hongkongensis* resists to ampicillin, ceftriaxone and erythromycin [64]. In our result, numerous multidrug resistance (MDR) genes were induced by temperature changes and consequently lead to higher efflux activity, indicating the external stimuli plays roles in drug resistance by regulating the efflux pumps expression. For example, the efflux pumps which are the homologs of *mexAB-oprM* and *acrAB-tolC* (LHK_02129-LHK_02131 and LHK_02825-LHK_02827 respectively) were upregulated at 20°C (Fig 6). Moreover, as shown our qRT-PCR result in Fig 8, two-fold enhancement of these genes with statistically significance were observed at fresh water temperature compared with human body temperature. On the contrary, no difference were found for other MDR genes such as two β-lactamase encoded genes (LHK_00876, LHK_03028) at both temperatures (S3 Fig).

Efflux pumps such as MexAB-OprM in *Pseudomonas aeruginosa* or AcrAB-TolC in *E*. *coli* belong to resistance nodulation division (RND) family are responsible for eliminating drugs, dyes and detergent from bacteria [65, 66]. They form a complex and highly coupled network with multiple mechanisms to transport substrate including bile acid, fatty acid and antibiotics and make bacteria become MDR [19, 67].

Since lower incubation temperature causes membrane solidification, fatty acid composition is altered to provide more short and branched fatty acid [68]. During this modification, certain metabolites and fatty acid components are in excess. Efflux pumps such as MexCD-OprJ in *Pseudomonas aeruginosa* are responsible for exporting membrane constituents in the fatty acid turnover process [69, 70]. The upregulation of AcrAB-TolC in *L*. *hongkongensis* at freshwater temperature possibly responsible for excluding redundant fatty acid components.

Temperature-regulated efflux pumps have been widely discovered in bacteria. For instance, increased efflux activity of *Pseudomonas fluorescens* (*P*. *fluorescens*) [71] and *Moraxella catarrhalis* [72] were measured at cooler temperature, suggesting the generosity of temperature-induced efflux pumps expression and activity. In *L*. *hongkongensis*, the transcription of efflux pumps elevated at freshwater temperature that may lead to the enhancement of drug resistance because of the increased efflux activity. Bacterial multidrug resistance have been a worldwide health issue in recent years. Investigation on temperature-induced drug resistance in other pathogens should be carried out to constrain this serious health problem.

## Conclusion

In this study, we performed transcriptomic analysis of *L*. *hongkongensis* at two physiologically relevant temperatures, 20°C and 37°C, mimicking the body temperature of fish and human respectively. Our result indicated the versatile and multi-dimensional adaptive mechanisms of *L*. *hongkongensis* in response to different external temperatures. In addition to the shift of metabolic equilibriums and over-expression of stress-related proteins induced by the temperature change, we found that numerous pathways were significantly altered. For example, genes of urease and anaerobic reductases were upregulated at human body temperature whereas, on the contrary, iron transport, branched amino acid transporter and RND efflux pumps were over-expressed at 20°C. In summary, our study demonstrated that the transcriptomic alteration in response to the temperature change and it provides the versatile adaptation to different host, as well as prepare *L*. *hongkongensis* for the challenge from the host immunity.

## Supporting Information

S1 Fig Mapping statistic of transcriptome data at 20°C and 37°C. Reads mapping to coding region (green), rRNA (blue), tRNA (grey) and non-coding RNA (orange) in genome at 20°C and 37°C RNA-seq.
(TIF)

S2 Fig Fold change correlation between RNA-seq and qRT-PCR. Data was plotted by log_2_ ratio from RNA-seq (x-axis) and qRT-PCR (y-axis). The transcript levels of qRT-PCR are expressed as means of three biological replicates.
(TIF)

S3 Fig The mRNA expression of gens related to beta-lactamases. Vertical error bars showed the standard deviations of biological triplicates. Expression level at 20°C was taken as reference for comparison. *P-value<0.05; **P-value<0.01.
(TIF)

S4 Fig Schematic diagram of the gene cassettes and expression pattern. The reads counts of each gene operons at 20°C (blue) and 37°C (red) were shown in IGV. Schematic organization of the cassettes was shown below the IGV.
(TIF)

S1 Table Primer used in this study. (PDF)

S2 Table Differential transcript and protein expression of *L*. *hongkongensis* at 20°C and 37°C. (PDF)

S1 Dataset List of genes found to be up-regulated at 37°C. (XLSX)

S2 Dataset List of genes found to be up-regulated at 20°C. (XLSX)
