# Supplementary figures and images for: Transcriptomic Analysis of Laribacter hongkongensis Reveals Adaptive Response Coupled with Temperature
Source: PLoS One. 2017 Jan 13;12(1):e0169998. doi: 10.1371/journal.pone.0169998 (PMC5234827; doi:10.1371/journal.pone.0169998)

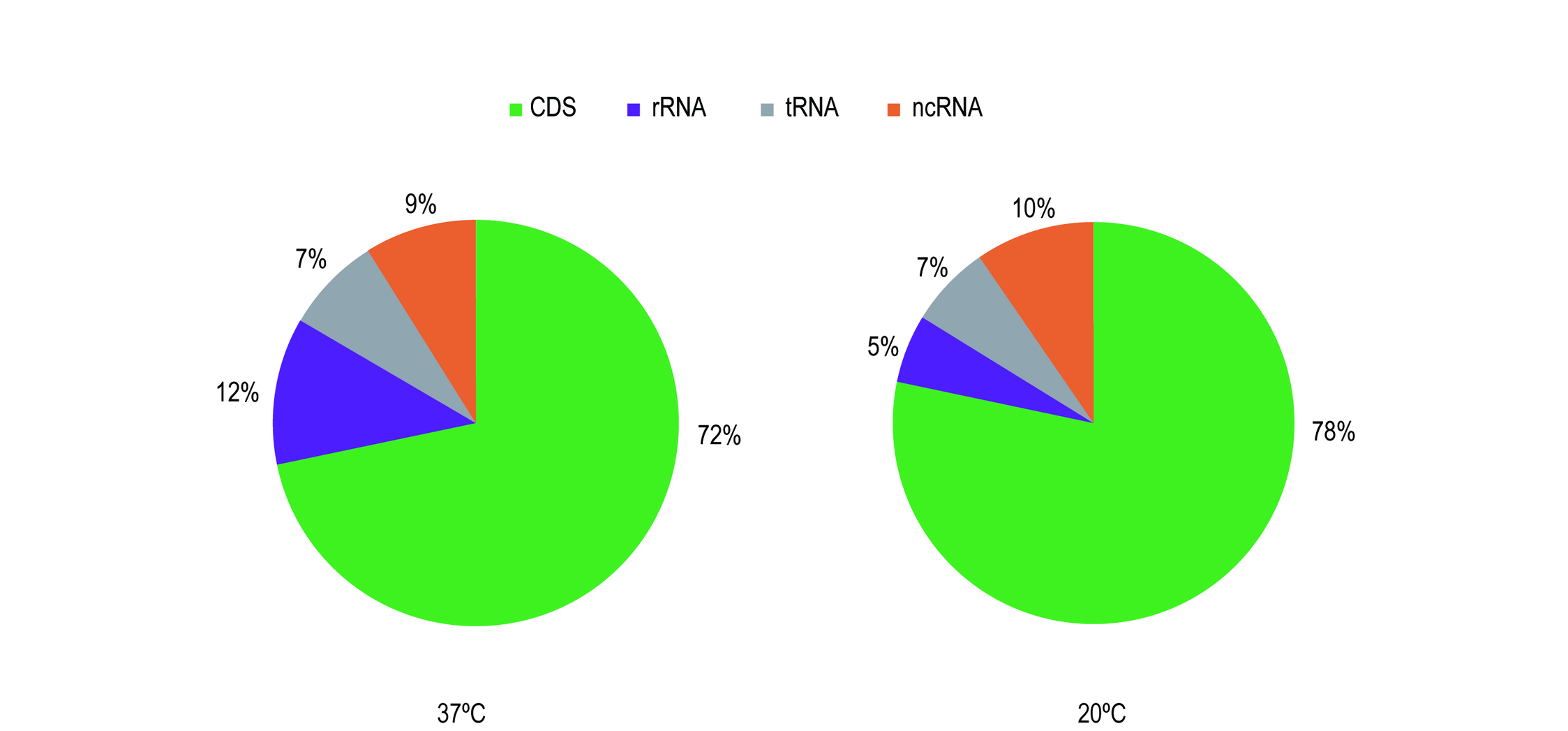

Supplement: S1 Fig — Reads mapping to coding region (green), rRNA (blue), tRNA (grey) and non-coding RNA (orange) in genome at 20°C and 37°C RNA-seq. (TIF) [file pone.0169998.s001.tif]

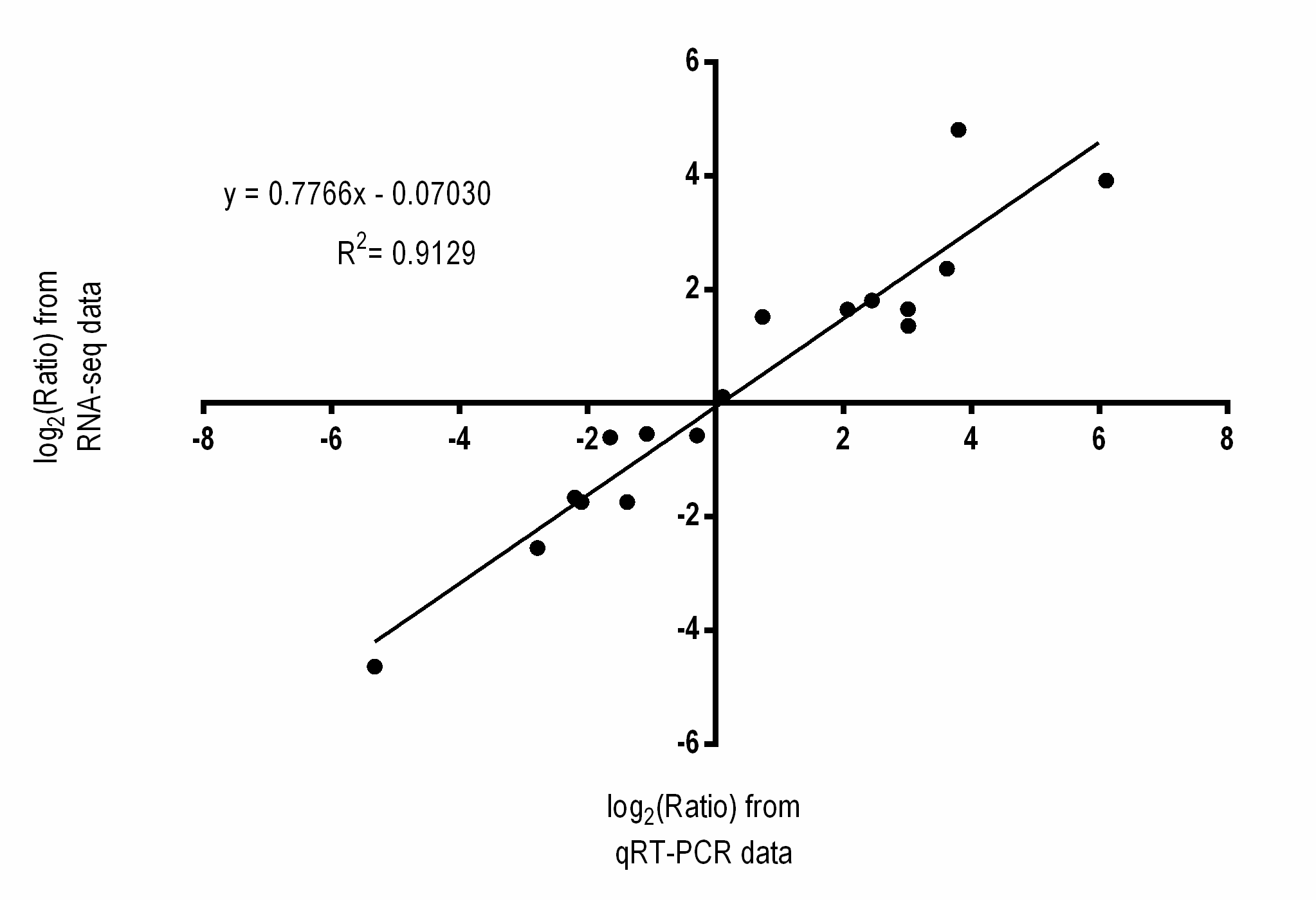

Supplement: S2 Fig — Data was plotted by log2 ratio from RNA-seq (x-axis) and qRT-PCR (y-axis). The transcript levels of qRT-PCR are expressed as means of three biological replicates. (TIF) [file pone.0169998.s002.tif]

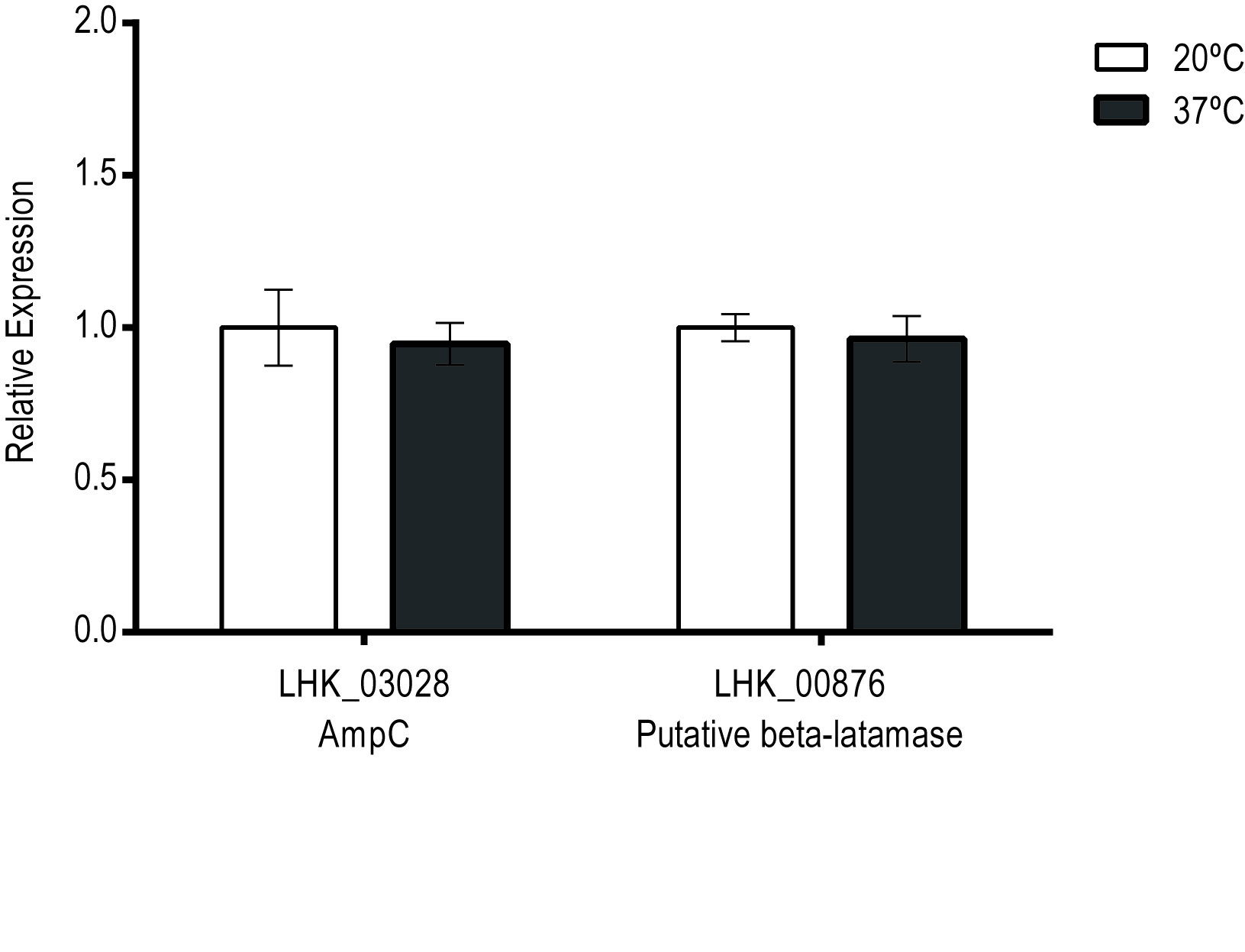

Supplement: S3 Fig — Vertical error bars showed the standard deviations of biological triplicates. Expression level at 20°C was taken as reference for comparison. *P-value<0.05; **P-value<0.01. (TIF) [file pone.0169998.s003.tif]

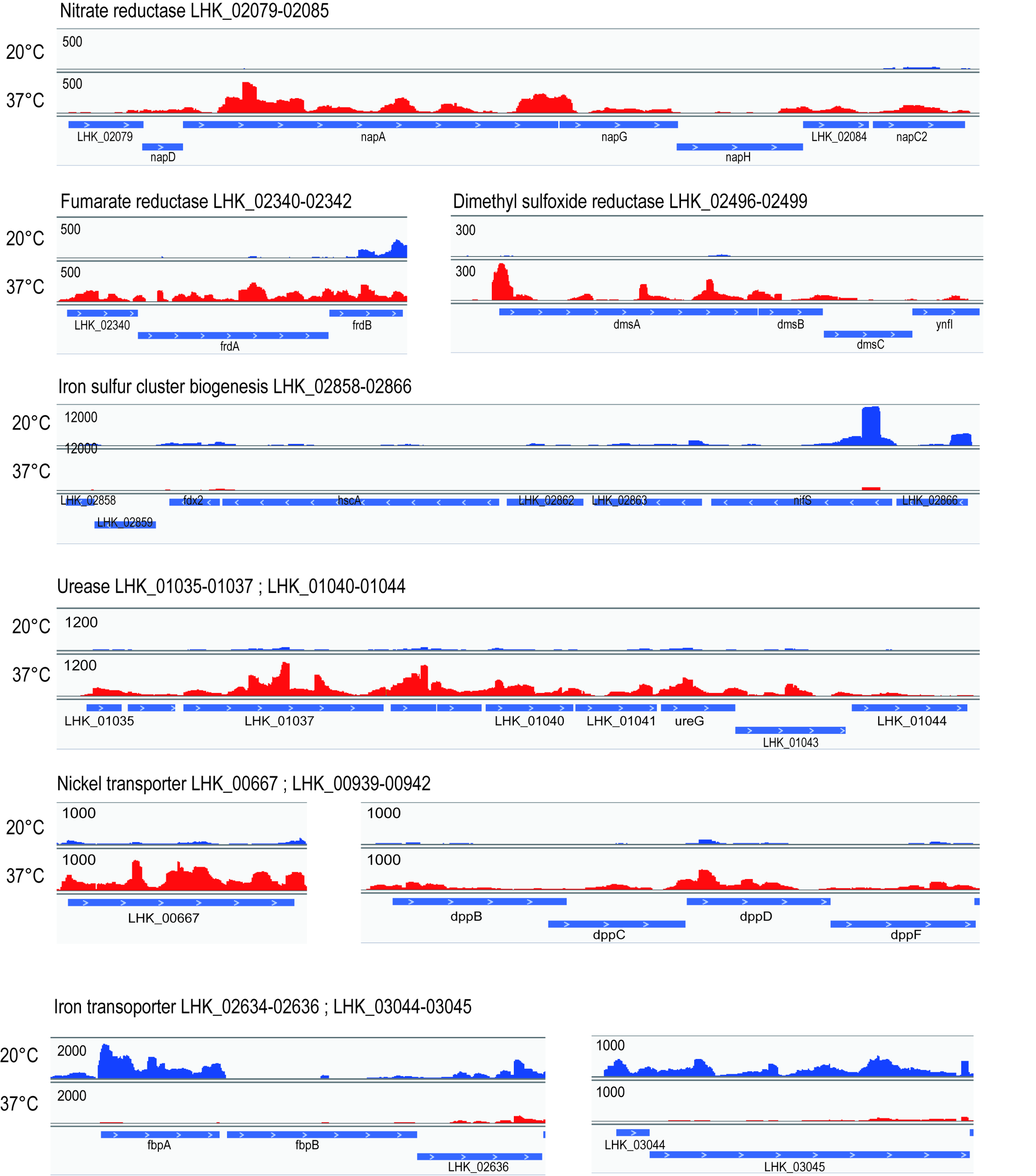

Supplement: S4 Fig — The reads counts of each gene operons at 20°C (blue) and 37°C (red) were shown in IGV. Schematic organization of the cassettes was shown below the IGV. (TIF) [file pone.0169998.s004.tif]
