# Supplementary material for: Transcriptomic Analysis of Laribacter hongkongensis Reveals Adaptive Response Coupled with Temperature
Source: PLoS One. 2017 Jan 13;12(1):e0169998. doi: 10.1371/journal.pone.0169998 (PMC5234827; doi:10.1371/journal.pone.0169998)
Supplement: S1 Table — (PDF) [file pone.0169998.s005.pdf]

S1 Table. Primers used in this study

Primer Name		Sequence (from 5' to 3')
LHK_00116	pat-F	CACGTCATCACCCGCTATAC
LHK_00116	pat-R	CCGTGTCGACATACCAGTCA
LHK_00246	rpoB-F	AGAACATGACCATCGCCTTC
LHK_00246	rpoB-R	TCGATGTGGATCGAGGTGTA
LHK_00499	exbB-F	AGTGGAGCCGGCAGTTCT
LHK_00499	exbB-R	GGTAATGGTCGAGTCCTTCG
LHK_00667	dppA-F	CAACCTCGGCTACATCACCT
LHK_00667	dppA-R	GTCGATGATGGCCTTCTTGT
LHK_00790	phos-F	AGTCGTACGCCTGCCAGTAG
LHK_00790	phos-R	CAGCTGTCGTTCAAGACCAA
LHK_00876	AmpR-F	AGTCGCAGATGTTCGGTTTC
LHK_00876	AmpR-R	GACAATGCAGCACCTCCAG
LHK_00932	cspA-F	CCGGTATCGTCAAATGGTTC
LHK_00932	cspA-R	GGACTGGATGGCAGAGAAGT
LHK_00939	dppB-F	GTTTTCGATGCCGATTTTCT
LHK_00939	dppB-R	GTCGTACATCAGGTCGATGC
LHK_00956	hem-F	GCTGGTGTTGCTGTACGTGA
LHK_00956	hem-R	GGCAATCAGCAGGTAAATGG
LHK_01006	tonB-F	GACCGGAACATCCCCTTG
LHK_01006	tonB-R	AAAACGTGGAAATCGACACC
LHK_01037	ureC-F	AAGACCTGCATCACCTTTGC
LHK_01037	ureC-R	CATGTCGTTCTTGGTGATGG
LHK_01042	ureG-F	GACAACCTGACGCTGACCTT
LHK_01042	ureG-R	GATGACCAGGATGTCGGACT
LHK_02081	napA-F	GAAGGACAGCAAGCTCAAGG
LHK_02081	napA-R	GGGTAAGGGTCGGAAACAAT
LHK_02129	mexA-F	TATGTCGACGTCACCCAGTC
LHK_02129	mexA-R	TCTTCCAGCTTGAGGCTGAC
LHK_02130	acrB-F	CGAAGACCAGGGAATGATGT
LHK_02130	acrB-R	TTCGTTTTCGAGGAAGTGCT
LHK_02131	nodT-F	CAGGACACCATCGACGAAC
LHK_02131	nodT-R	TGTCGATACCCTTGTCAAAGC
LHK_02132	tetR-F	TTCTCCAGCAGGGCTATCAC
LHK_02132	tetR-R	GCAGCGATGATCTCTTCCTT

LHK_02337	argB-37-F	ATGATTCCCAAGGTGCAGTC
LHK_02337	argB-37-R	GTCAGGATTTCCAGCAGCAG
LHK_02341	frdA-F	ACGTCTACACCCTGCGTCAT
LHK_02341	frdA-R	TGCTGCGCTGCAATAATATC
LHK_02634	fbpA-F	ATTCCAGGACACTGGAGCAG
LHK_02634	fbpA-R	GTGCGGGTGTTGAAGAAGAT
LHK_02651	col-F	TCGATGAAGGACAACAACCA
LHK_02651	col-R	ACGTAACCGGCATCCTTGTA
LHK_02735	RTX-F	GACATCCTGATCGGTGGTTC
LHK_02735	RTX-R	TCAGGTGGAAATCCTTGACC
LHK_02825	natC-F	CGACCTCGAAGCTCTTTTTG
LHK_02825	natC-R	CGTATTTTGACGTGCTGCTG
LHK_02826	acrB-F	AACCAGGATAACGCTGTTGC
LHK_02826	acrB-R	TACCCTGTCGCTGATCCTGT
LHK_02827	acrA-F	CGAAAGCTCTGCTTCGATTC
LHK_02827	acrA-R	AAGAAGAAACCCGTCCGGTA
LHK_02828	acrR-F	GGACCACGCTCTTGAAGATT
LHK_02828	acrR-R	GCCACCAAGATCGAACACAT
LHK_02829	argB-20-F	CGTCAACAAGGAAATCGTCA
LHK_02829	argB-20-R	CCGTCCTGGATGTAGAGCTT
LHK_02918	RTX-F	ACATCCAGTCTACGGCAACC
LHK_02918	RTX-R	GCTTCATTTCAATCCGCAAT
LHK_03028	AmpC-F	GAAACCTACGGCGTCAAGTC
LHK_03028	AmpC-R	CTGTAATGCCGACTGCAATG
