# Supplementary material for: Transcriptomic Analysis of Laribacter hongkongensis Reveals Adaptive Response Coupled with Temperature
Source: PLoS One. 2017 Jan 13;12(1):e0169998. doi: 10.1371/journal.pone.0169998 (PMC5234827; doi:10.1371/journal.pone.0169998)
Supplement: S2 Table — (PDF) [file pone.0169998.s006.pdf]

S2 Table. Differential transcript and protein expression of *L. hongkongensis* at 20°C and 37°C.

	*Fold change 20°C / 37°C	
Gene	Proteome	Transcriptome
LHK_02829	2.5	3.12
LHK_01819	2.41	12.75
LHK_00236	2.04	-1.82
LHK_00011	Only express at 20°C	3.28
LHK_01152	Only express at 20°C	13.20
LHK_01472	Only express at 20°C	1.11
LHK_01018	Only express at 20°C	-2.72
LHK_02337	-2.09	-8.06
LHK_02119	-3.06	-1.53
LHK_02507	Only express at 37°C	-1.63
LHK_03194	Only express at 37°C	1.44

* Negative number represents reverse ratio of fold change.
